# Risk Factors and Nonlinear Risk Patterns of Prolonged Air Leak After Robot-Assisted Lung Resection for Lung Cancer: A Retrospective Cohort Study

**DOI:** 10.3390/cancers18101612

**Published:** 2026-05-15

**Authors:** Hao Xu, Han Zhang, Linyou Zhang

**Affiliations:** Department of Thoracic Surgery, The Second Affiliated Hospital of Harbin Medical University, Harbin 150086, China; xuhao@hrbmu.edu.cn (H.X.); hanzhang@hrbmu.edu.cn (H.Z.)

**Keywords:** prolonged air leak, robot-assisted thoracic surgery, lung cancer, restricted cubic spline, nomogram

## Abstract

**Background/Objectives:** Prolonged air leak (PAL) remains a common complication after lung resection and may delay postoperative recovery and subsequent treatment. This study aimed to identify clinical factors associated with PAL after robot-assisted thoracic surgery (RATS) and to explore potential nonlinear relationships using restricted cubic spline (RCS) modeling. **Methods:** A retrospective cohort of 1185 patients who underwent RATS for primary lung cancer was analyzed. Multivariable Firth logistic regression was used to identify independent predictors of PAL (≥5 days). A nomogram was constructed based on the final model and internally validated using 1000 bootstrap resamples; its clinical utility was assessed using decision curve analysis. RCS analysis was performed to evaluate potential nonlinear associations. **Results:** A total of 98 patients (8.3%) developed PAL. Male sex was independently associated with increased PAL risk (OR 3.29, *p* < 0.001), whereas higher FEV1 was associated with reduced risk (OR 0.50 per 1-L increase, *p* < 0.001). BMI showed a modest protective effect (OR 0.91, *p* = 0.01). Age was not significant in the linear model (*p* = 0.86), but RCS analysis demonstrated a significant nonlinear association, with increased risk at older ages. The nomogram demonstrated moderate discrimination (apparent C-statistic 0.670, optimism-corrected 0.644) and good calibration, with decision curve analysis confirming net clinical benefit over treat-all and treat-none strategies. **Conclusions:** Male sex and impaired pulmonary function are key predictors of PAL after RATS. Nonlinear modeling revealed complex age-related risk patterns not captured by conventional approaches. The proposed nomogram may assist in preoperative risk stratification and perioperative decision-making.

## Simple Summary

Prolonged air leak (PAL) is a common complication after robotic lung surgery that can delay recovery and subsequent cancer treatment. In this study of 1185 patients undergoing robot-assisted thoracic surgery (RATS) for lung cancer, we identified male sex and reduced pulmonary function (measured by FEV1) as the strongest predictors of PAL. While age alone was not a significant linear predictor, advanced statistical modeling revealed a nonlinear risk increase after approximately 70 years of age. We developed a nomogram—a simple graphical tool—that integrates these factors to estimate each patient’s individual risk of PAL before surgery. This tool showed moderate discrimination and clinical utility, potentially helping surgeons identify high-risk patients who may benefit from preventive interventions or modified perioperative management.

## 1. Introduction

Lung cancer remains the leading cause of cancer-related mortality worldwide, and surgical resection remains the cornerstone of curative treatment for early-stage disease [1]. Prolonged air leak (PAL), typically defined as an air leak persisting beyond 5 postoperative days, is one of the most common complications following pulmonary resection [2]. The occurrence of PAL has been associated with prolonged hospital stay, increased postoperative complications, additional interventions, and higher healthcare costs [3]. Given these clinical and economic burdens, accurate preoperative risk stratification for PAL has become increasingly important to guide surgical decision-making and perioperative management [4].

PAL has been extensively investigated, with numerous efforts to identify risk factors and develop predictive models for patients undergoing pulmonary resection [5,6]. These models typically incorporate demographic characteristics, pulmonary function, imaging findings, and operative variables to estimate PAL risk [7]. However, most existing models were developed in heterogeneous surgical populations and were based on conventional linear modeling assumptions. Importantly, relatively few studies have specifically examined patients undergoing robotic-assisted thoracic surgery (RATS), despite the increasing adoption of this technique in lung cancer surgery [8,9].

The present study aimed to identify predictors of prolonged air leak following robot-assisted thoracic surgery (RATS) and to evaluate whether restricted cubic spline modeling could provide additional risk information beyond conventional approaches. Based on these analyses, we developed and internally validated a predictive model to support individualized preoperative risk assessment.

## 2. Materials and Methods

This retrospective cohort study was conducted in accordance with the STROBE statement. The study protocol was approved by the Ethics Committee of the Second Affiliated Hospital of Harbin Medical University (Approval No. KY2025-103). The requirement for informed consent was waived due to the retrospective study design. All procedures were conducted in accordance with the Declaration of Helsinki.

### 2.1. Surgical Procedure

Surgical procedures were performed using the da Vinci Xi robotic surgical system (Intuitive Surgical, Sunnyvale, CA, USA) via a standardized three-arm approach. Anatomical lobectomy or segmentectomy was performed according to standard thoracic surgical principles, with parenchymal, bronchial, and vascular division performed using endoscopic linear staplers. A 28-Fr chest tube was routinely placed at the end of the surgery and removed according to institutional protocols once no air leak was detected and drainage was <100 mL per 24 h.

All procedures were performed under general anesthesia with single-lung ventilation. Intraoperative air leak testing was performed by submerging the resected lobe in saline solution after stapler division to assess parenchymal integrity; however, results were not systematically recorded and could not be incorporated as a model variable. Pleural sealants and stapler buttressing materials were used at the discretion of the operating surgeon and were likewise not systematically documented, representing a limitation of this retrospective analysis. Cases were performed by attending thoracic surgeons credentialed for robotic surgery at the institution, using a consistent procedural framework throughout the study period.

### 2.2. Study Population

Patients who underwent RATS for primary lung cancer at the Second Affiliated Hospital of Harbin Medical University between January 2020 and December 2024 were retrospectively screened for eligibility. Inclusion criteria were histopathologically confirmed primary lung cancer, clinical stage I–III NSCLC according to the 8th edition AJCC staging system, anatomical lobectomy or segmentectomy, and availability of complete perioperative clinical and pulmonary function measurements. Exclusion criteria included prior pulmonary resection, synchronous malignancy, receipt of neoadjuvant therapy, and missing key clinical or outcome data. A total of 1185 patients met the inclusion criteria and were included in the final analysis.

### 2.3. Data Collection

Baseline variables included demographic characteristics (age, sex, body mass index [BMI], and smoking history) and comorbidities (hypertension, diabetes, coronary artery disease [CAD], and cerebrovascular disease [CVA]). Preoperative pulmonary function parameters included forced expiratory volume in 1 s (FEV1), FEV1/FVC ratio, maximal voluntary ventilation (MVV), and diffusing capacity for carbon monoxide (DLCO). Comorbidity burden was assessed using a simplified comorbidity score derived from diabetes mellitus and cerebrovascular disease components available in the dataset—hereafter referred to as the modified CCI—which represents only a partial approximation of the full Charlson Comorbidity Index, as other standard CCI domains were not consistently documented in this retrospective dataset [10]. Perioperative outcomes included operative time, estimated blood loss, duration of chest tube placement, postoperative length of stay, and the occurrence of PAL. Among pulmonary function variables, FEV1 was selected as the primary variable for regression analyses based on its established clinical relevance.

### 2.4. Outcome Definition

The primary outcome was PAL, defined as a persistent postoperative air leak lasting 5 days or more, in accordance with the definition proposed by the European Society of Thoracic Surgeons (ESTS) [5]. A sensitivity analysis using a more stringent threshold of 7 days or more was also performed to assess the robustness of findings.

### 2.5. Statistical Analysis

Continuous variables were summarized as mean ± SD or median (IQR), and categorical variables as frequency and percentage. Comparisons between age-stratified groups were performed using the Kruskal–Wallis test for continuous variables and the chi-square or Fisher’s exact test, as appropriate, for categorical variables.

Independent predictors of PAL were identified using multivariable Firth’s penalized logistic regression. Continuous predictors were initially modeled as linear terms. This approach reduces small-sample bias and improves the stability of parameter estimates. The events-per-variable ratio exceeded 10, supporting model stability. The multivariable model included age, sex, BMI, modified CCI, FEV1, smoking history, and surgical procedure type. Multicollinearity among predictors was assessed using variance inflation factors (VIFs), and all VIF values were <3.

Restricted cubic spline (RCS) modeling was used to evaluate potential nonlinear relationships between continuous predictors and PAL risk. Three knots were placed at the 5th, 50th, and 95th percentiles (Age: 42, 60, 73 years; FEV1: 1.52, 2.38, 3.49 L; BMI: 19.0, 23.8, 29.5 kg/m^2^). A 3-knot specification was chosen because it is the recommended default for exploratory nonlinearity assessment with this sample size, consuming only 2 degrees of freedom per predictor and preserving model stability given the available events-per-variable ratio. To confirm that findings were not sensitive to knot number, a 4-knot sensitivity analysis was also performed (Supplementary Table S1). Nonlinearity was assessed using likelihood ratio tests comparing spline and linear models, and model fit was evaluated using the Akaike information criterion (AIC). Sensitivity analyses included fully adjusted models, an alternative PAL threshold (≥7 days), and a 4-knot RCS specification to assess sensitivity to knot selection.

To further examine the relationship between age and PAL, a stepwise attenuation analysis was performed. Covariates were sequentially added to an age-only model, and changes in the age coefficient were used to quantify attenuation of the association.

A nomogram was constructed based on the multivariable model to estimate individualized PAL risk. Model discrimination was assessed using the C-statistic with 95% confidence intervals obtained from 1000 bootstrap samples. An optimism-corrected C-statistic was also reported. Model calibration was evaluated using the Hosmer-Lemeshow test and bootstrap-corrected calibration plots.

Decision curve analysis (DCA) was performed to evaluate net benefit across a range of threshold probabilities (0–30%), compared with the treat-all and treat-none strategies. A model including age alone was also evaluated for comparison.

All statistical analyses were performed using R version 4.5.2 (R Foundation for Statistical Computing, Vienna, Austria). For comparison with existing scoring systems, variables from the Pompili ESTS score (male sex, FEV1, and BMI) and the Brunelli score (age, FEV1/FVC ratio, and BMI) were refitted using logistic regression in the study cohort. Discrimination was compared using DeLong’s test for correlated ROC curves. A two-tailed *p* value < 0.05 was considered statistically significant.

## 3. Results

### 3.1. Baseline Characteristics

A total of 1185 patients with primary lung cancer underwent RATS. Patients were categorized into three age groups: <60 years (*n* = 549), 60–69 years (*n* = 457), and ≥70 years (*n* = 179). Baseline characteristics are summarized in Table 1.

The proportion of male patients increased progressively with age (34.1%, 39.6%, and 46.9% in the <60, 60–69, and ≥70 years groups, respectively; *p* = 0.006). Similarly, the prevalence of ever smoking increased with age (13.1%, 21.7%, and 22.9%, respectively; *p* < 0.001). BMI did not differ significantly among age groups (*p* = 0.70).

Pulmonary function parameters declined with age. Mean FEV1 decreased from 2.7 L in patients < 60 years to 2.1 L in those ≥70 years (*p* < 0.001), with similar trends observed for FEV1/FVC and DLCO (both *p* < 0.001). Older patients also had higher rates of hypertension and diabetes (both *p* < 0.001), whereas coronary artery disease did not differ significantly among age groups (*p* = 0.168).

Lobectomy was more frequently performed in older patients (44.3%, 57.3%, and 67.0%, respectively; *p* < 0.001), whereas segmentectomy was more common in younger patients. Tumor size also increased with age (*p* < 0.001).

### 3.2. Age and Perioperative Outcomes

**Figure 1:**
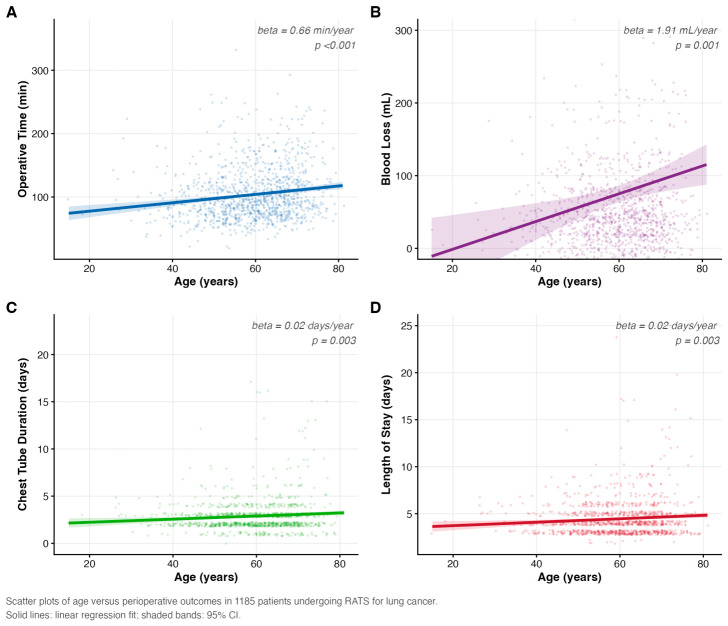
Association between age and perioperative outcomes following robot-assisted thoracic surgery (RATS). Scatter plots showing the relationships between age and perioperative outcomes: (**A**) operative time, (**B**) intraoperative blood loss, (**C**) chest tube duration, and (**D**) postoperative length of stay. Each point represents an individual patient (jittered for clarity). Solid lines represent linear regression fits with shaded 95% confidence intervals. Beta coefficients (per 1-year increment in age) and corresponding *p* values are annotated in each panel.

Increasing age was associated with longer chest tube duration and postoperative length of stay (both beta = 0.02 days/year; *p* = 0.003). Operative time and intraoperative blood loss also showed modest but statistically significant linear increases with age (beta = 0.66 min/year and beta = 1.91 mL/year, respectively; both *p* ≤ 0.001) (Figure 1).

### 3.3. Multivariable Analysis of Risk Factors for Prolonged Air Leak

Among the 1185 patients, 98 events of prolonged air leak (PAL ≥ 5 days)occurred. Multivariable Firth’s penalized logistic regression identified independent predictors of PAL (Table 2).

Male sex was strongly associated with increased PAL risk (OR 3.29, 95% CI 1.96–5.54, *p* < 0.001). In contrast, higher FEV1 was independently associated with lower odds of PAL (OR 0.50 per 1-L increase, *p* < 0.01). Higher BMI was also associated with lower PAL risk (OR 0.91, 95% CI 0.85–0.98, *p* = 0.01). Age was not independently associated with PAL (OR 1.00, 95% CI 0.97–1.02, *p* = 0.86). Smoking history and modified Charlson Comorbidity Index were likewise not significant predictors. Lobectomy demonstrated borderline significance compared with segmentectomy (OR 1.30, *p* = 0.24).

### 3.4. Nonlinear Associations Between Continuous Predictors and Prolonged Air Leak

**Figure 2:**
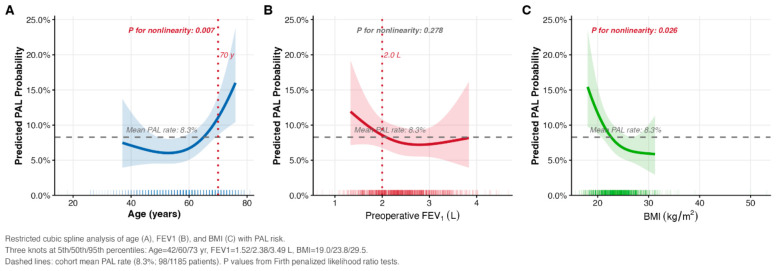
Restricted cubic spline analysis of nonlinear associations between continuous predictors and prolonged air leak. Restricted cubic spline (RCS) curves showing the predicted probability of prolonged air leak (PAL) across the observed range of (**A**) age, (**B**) preoperative FEV1, and (**C**) BMI. Three knots were placed at the 5th, 50th, and 95th percentiles of each variable (Age: 42, 60, 73 years; FEV1: 1.52, 2.38, 3.49 L; BMI: 19.0, 23.8, 29.5 kg/m^2^). Shaded areas represent 95% confidence intervals. Dashed horizontal lines indicate the cohort mean PAL rate (8.3%; 98/1185 patients). Dotted vertical reference lines indicate clinically relevant thresholds (age 70 years; FEV1 2.0 L). *p* values for nonlinearity are derived from likelihood ratio tests comparing the spline model to the corresponding linear model, using Firth’s penalized logistic regression. The age model was adjusted for BMI; the FEV1 and BMI models were adjusted for age. Abbreviations: BMI, body mass index; FEV1, forced expiratory volume in 1 s; PAL, prolonged air leak; RCS, restricted cubic spline.

Restricted cubic spline (RCS) analysis was performed to assess potential nonlinear relationships between age, FEV1, BMI, and PAL risk (Table 3). In the age-adjusted analysis, age demonstrated a significant nonlinear association with PAL (*p* for nonlinearity = 0.007). BMI also demonstrated significant nonlinearity (*p* = 0.026), whereas FEV1 did not (*p* = 0.278). These findings remained consistent after full adjustment, with age and BMI retaining significant nonlinear associations, whereas FEV1 remained linear. Using the more stringent PAL definition (≥7 days) yielded consistent results, with age remaining significantly nonlinear (*p* = 0.011), and BMI also showing significant nonlinearity (*p* = 0.043). To assess sensitivity to knot selection, a 4-knot RCS analysis was additionally performed; the nonlinear association of age with PAL risk remained statistically significant (*p* = 0.009), supporting the robustness of this finding (Supplementary Table S1). The corresponding spline curves are shown in Figure 2.

### 3.5. Stepwise Attenuation Analysis

This analysis was performed specifically to explain the apparent discrepancy between the non-significant linear age effect in the multivariable model (*p* = 0.86) and the significant nonlinear association identified by RCS (*p* = 0.007), and is not intended as a standard component of prediction model development.

In the stepwise attenuation analysis, the crude association between age and PAL risk was weak and non-significant (OR 1.011 per year, 95% CI 0.988–1.035, *p* = 0.374). Sequential adjustment for FEV1 attenuated the age coefficient by 54.2%. Upon further adjustment for sex, the age coefficient crossed the null (OR 0.983), a pattern consistent with sex functioning as a suppressor variable rather than a confounder in this context. In the fully adjusted model, the age coefficient remained non-significant (OR 0.980, 95% CI 0.954–1.007, *p* = 0.142), consistent with the primary multivariable analysis. These findings indicate that the age-PAL association is substantially mediated by pulmonary function and sex distribution, and that the significant nonlinear threshold effect identified by RCS analysis represents an effect operating primarily beyond approximately 70 years of age.

### 3.6. Nomogram and Clinical Utility of the Prediction Model

**Figure 3:**
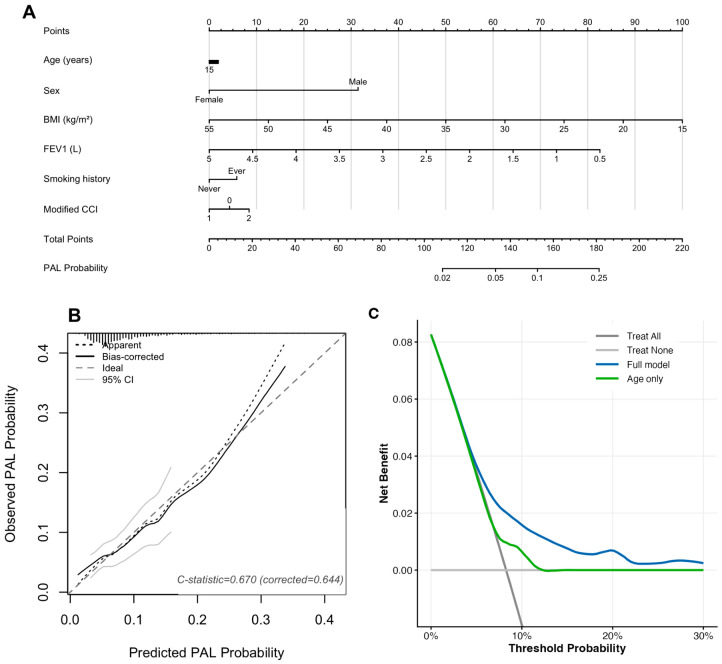
Nomogram for predicting prolonged air leak, calibration curve, and decision curve analysis. (**A**) Nomogram based on the multivariable Firth penalized logistic regression model for individualized preoperative prediction of prolonged air leak (PAL) after robot-assisted thoracic surgery. To use the nomogram, locate each variable on its respective axis, draw a vertical line to the Points scale, sum the total points, and project to the PAL Probability axis. (**B**) Bootstrap-corrected calibration curve (1000 resamples) showing agreement between predicted and observed PAL probability. The dotted line represents apparent calibration; the solid line represents bias-corrected calibration; the dashed diagonal line represents ideal calibration. The apparent C-statistic was 0.670 (bias-corrected: 0.644). (**C**) Decision curve analysis showing the net clinical benefit of the full multivariable model compared with an age-only model, treat-all, and treat-none strategies across threshold probabilities of 0–30%. Abbreviations: CCI, Charlson Comorbidity Index; FEV1, forced expiratory volume in 1 s; PAL, prolonged air leak.

A nomogram was constructed to estimate individualized PAL risk (Figure 3). Decision curve analysis showed that the model provided greater net benefit than the treat-all and treat-none strategies across clinically relevant threshold probabilities and outperformed an age-only model.

### 3.7. Subgroup Analysis

**Figure 4:**
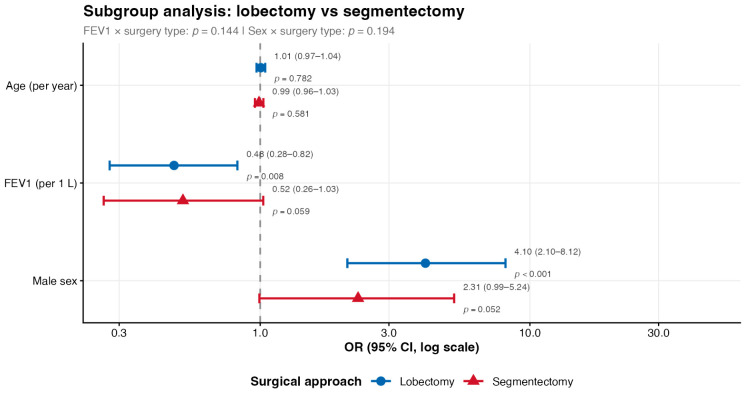
Subgroup analysis of risk factors for prolonged air leak by surgical approach. Forest plot showing odds ratios (OR) and 95% confidence intervals for FEV1, male sex, and age in patients undergoing lobectomy (n = 625, blue circles) and segmentectomy (n = 560, red triangles), estimated using Firth’s penalized logistic regression. The x-axis is presented on a logarithmic scale. *p* values for the interaction between surgical type and each predictor are displayed in the subtitle (FEV1 × surgery type: *p* = 0.144; sex × surgery type: *p* = 0.194). No significant interactions were identified, supporting the consistency of these risk factor associations across surgical approaches. Abbreviations: CI, confidence interval; FEV1, forced expiratory volume in 1 s; OR, odds ratio.

Subgroup analyses were performed separately in patients undergoing lobectomy (*n* = 625) and segmentectomy (*n* = 560). FEV1 and male sex remained the dominant predictors in both subgroups, with consistent directions of association. In the lobectomy subgroup, FEV1 was associated with lower PAL risk (OR 0.48, 95% CI 0.28–0.82, *p* = 0.008) and male sex with higher risk (OR 4.10, 95% CI 2.10–8.12, *p* < 0.001). Consistent findings were observed in the segmentectomy subgroup (FEV1: OR 0.52, 95% CI 0.26–1.03, *p* = 0.059; male sex: OR 2.31, 95% CI 0.99–5.24, *p* = 0.052). Formal interaction testing demonstrated no significant interaction between surgical type and FEV1 (*p* = 0.144) or sex (*p* = 0.194), indicating that the identified risk factors operated consistently regardless of surgical approach (Figure 4).

### 3.8. Risk Stratification

Based on the predicted PAL probabilities from the multivariable model, patients were classified into three risk strata using thresholds of 5% and 10% (Supplementary Table S2). The low-risk group (predicted probability < 5%) comprised 375 patients (31.6%) with an observed PAL rate of 4.3%. The intermediate-risk group (5–10%) included 495 patients (41.8%) with a PAL rate of 6.5%. The high-risk group (>10%) comprised 315 patients (26.6%) with a PAL rate of 15.9%. PAL rates differed significantly across the three strata (*p* < 0.001). The primary utility of this stratification is to identify patients warranting heightened perioperative vigilance and individualized counseling; the absolute PAL rate of 15.9% in the high-risk group indicates that most patients in this stratum will not experience PAL, and this tool should not be used to justify high-risk prophylactic interventions in isolation.

### 3.9. Comparison with Existing Scoring Systems

To further evaluate the discriminative performance of the model, comparisons were performed with two widely used PAL prediction variable sets, namely the Pompili ESTS score and the Brunelli score, refitted in the study cohort. When variables from the Pompili ESTS score and Brunelli score were refitted, the resulting AUCs were 0.627 and 0.611, respectively. The present model demonstrated significantly superior discrimination (AUC 0.689) compared with both the Pompili variable set (ΔAUC = 0.062, *p* = 0.006) and the Brunelli variable set (ΔAUC = 0.078, *p* = 0.019) using DeLong’s test. It should be noted that this comparison does not constitute external validation of the original published scoring systems. The Pompili ESTS and Brunelli variable sets were re-fitted using logistic regression in the present cohort; the resulting AUCs reflect the discriminative capacity of each variable set within this population rather than the performance of the original score algorithms. A true external validation using original published coefficients was not feasible due to differences in variable definitions across studies.

## 4. Discussion

This study aimed to develop a clinically applicable preoperative risk stratification model for prolonged air leak (PAL) following robot-assisted thoracic surgery (RATS) for lung cancer, with a focus on potential nonlinear relationships assessed using restricted cubic spline (RCS) modeling.

Male sex was strongly associated with an increased risk of PAL, whereas higher preoperative FEV1 was associated with a lower risk. Age was not an independent predictor in the multivariable model. However, RCS analysis revealed a significant nonlinear association between age and PAL risk. This finding suggests that conventional linear models may not fully capture age-related risk patterns. From a clinical perspective, this finding suggests that age-related risk should not be interpreted as a simple linear increase and that patients at older ages may require more individualized perioperative risk assessment.

A nomogram was developed based on these variables to support individualized preoperative risk estimation. Decision curve analysis indicated potential clinical utility. Overall, these results highlight the importance of considering nonlinear relationships when evaluating PAL risk after robotic lung resection.

PAL after pulmonary resection has been widely studied, and many prediction models have been proposed. Early models focused on pulmonary function, BMI, and surgical factors [11,12,13]. Later studies used larger datasets and included more clinical variables [14,15,16,17,18,19,20]. More recent work has explored machine learning and other flexible modeling approaches [21,22,23,24]. These studies consistently show that pulmonary function, BMI, and surgical factors are key predictors of PAL [7,8].

However, most existing models were developed in mixed surgical populations. They also assume linear relationships between predictors and outcomes. Importantly, the present study focused on a relatively homogeneous cohort of patients undergoing RATS. This reduces variability related to surgical approach and allows a more consistent evaluation of risk patterns.

Among existing approaches, the Pompili ESTS score and Brunelli score are widely used in clinical practice. In comparison, the present model showed improved discrimination in this RATS cohort. This suggests that incorporating nonlinear effects may provide additional predictive value beyond traditional approaches.

A key observation in this study was the behavior of age. Age was not significant in the multivariable model. In contrast, RCS analysis demonstrated a nonlinear association with PAL risk, with a more pronounced increase at older ages. This pattern indicates that the effect of age is not uniform across its range.

Age is closely related to pulmonary function and sex distribution. Both are established risk factors for PAL [25,26]. After adjusting for FEV1 and sex, the association between age and PAL was substantially attenuated. This suggests that part of the age effect is explained by these variables.

However, a residual nonlinear pattern remained after adjustment. This finding suggests that additional factors may contribute to PAL risk. These factors may not be fully captured by routinely measured clinical variables. Possible explanations include subclinical emphysema, changes in lung structure, or impaired tissue healing capacity.

The associations of FEV1 and sex with PAL risk are consistent with previous studies. Reduced pulmonary function may reflect impaired lung compliance and diminished parenchymal healing capacity. These factors increase the risk of persistent air leak. The higher risk observed in male patients may be related to a greater burden of smoking-related structural changes in the lung. Differences in lung anatomy and physiology may also play a role.

The nomogram provides a practical tool for clinical use. It uses variables that are available before surgery and allows estimation of individual PAL risk. Decision curve analysis showed that the model provides greater net benefit than treat-all and treat-none strategies across clinically relevant threshold probabilities. This suggests that the model may assist in perioperative decision-making, including surgical planning and postoperative management.

The model showed consistent performance across different surgical approaches. The effects of FEV1 and sex were similar in both lobectomy and segmentectomy. No significant interaction with surgical type was observed. In addition, risk stratification showed a clear gradient, with higher predicted risk corresponding to higher observed PAL rates. This supports the model’s ability to distinguish clinically meaningful risk levels.

This study has several strengths. It included a relatively large cohort of patients undergoing RATS, allowing focused evaluation in a contemporary minimally invasive setting. RCS modeling was used to explore nonlinear relationships that may be overlooked in conventional analyses. The clinical utility of the model was also assessed using decision curve analysis.

The age spline curve (Figure 2A) shows that predicted PAL probability remains relatively stable near the cohort mean below approximately 60 years of age and rises progressively thereafter, with the most pronounced acceleration beyond 70 years. Clinically, this nonlinear pattern suggests that age-related PAL risk is not a uniform linear phenomenon but rather a threshold effect concentrated in the oldest surgical candidates. For patients aged 70 years or older—who in this cohort had a lobectomy rate of 67.0% and an observed PAL rate of 14.5%—heightened preoperative counseling and consideration of risk-mitigation strategies may be particularly warranted. This pattern likely reflects the cumulative effects of subclinical emphysema, reduced parenchymal compliance, and impaired tissue healing that accumulate disproportionately in advanced age and are not fully captured by FEV1 alone. Importantly, this nonlinear association should be regarded as an exploratory finding: although statistically robust across 3-knot (*p* = 0.007) and 4-knot (*p* = 0.009) specifications, the wide confidence intervals at older ages reflect substantial uncertainty, and prospective validation is required before this threshold is used to guide clinical decisions.

The preoperative predictors identified in this study likely act as upstream risk modifiers that increase susceptibility to PAL when intraoperative conditions are unfavorable. Male patients with reduced FEV1 may have greater underlying emphysematous parenchymal change, rendering them less tolerant of incomplete fissure development or stapler line tension. In such patients, intraoperative findings of dense pleural adhesions or incomplete fissures may compound the preoperative risk profile, potentially producing a multiplicative rather than additive effect on PAL probability. The modest C-statistic of 0.644 reflects the inherent performance ceiling of preoperative-only prediction: PAL is substantially determined by intraoperative factors—including fissure completeness, pleural adhesion burden, and parenchymal staple line integrity—none of which are measurable before surgery. This limitation is shared by all existing preoperative PAL nomograms and underscores the need for future studies integrating both preoperative and intraoperative data in a unified prediction framework.

For patients classified as high-risk (predicted PAL probability > 10%), several perioperative considerations may be appropriate based on existing evidence and clinical reasoning. These include preoperative counseling regarding the likelihood of extended chest tube duration and hospital stay; discussion of prophylactic use of pleural sealants or stapler buttressing materials, particularly in patients with emphysematous parenchyma or incomplete fissures identified on preoperative CT; consideration of water-seal rather than suction chest tube management protocols; and heightened postoperative physiotherapy surveillance. We emphasize that these recommendations are based on clinical reasoning and existing literature rather than direct evidence from this study, and that prospective evaluation of risk-stratified management protocols is needed to determine whether targeted interventions reduce PAL incidence in predicted high-risk patients.

The present model relied on FEV1 expressed in absolute liters, which does not adjust for age, sex, or body habitus and may reduce generalizability across demographically diverse populations. Future studies should incorporate FEV1% predicted and predicted postoperative FEV1 (ppoFEV1), which provide physiologically normalized measures of pulmonary reserve. Additionally, DLCO was collected in this cohort (Table 1) but was excluded from the primary model to avoid multicollinearity with FEV1 and to preserve parsimony. DLCO % predicted may capture aspects of parenchymal membrane integrity independent of airflow limitation and could contribute meaningfully to model discrimination in future work. A comprehensive pulmonary function profile combining FEV1% predicted, DLCO % predicted, and ppoFEV1 represents a priority direction for model refinement.

This study has several important limitations. First, as a retrospective single-center analysis, it is subject to selection bias and institutional referral patterns that may not generalize to other settings. Second, several variables known to strongly influence PAL—including CT-based emphysema grading, fissure completeness, intraoperative air leak severity, pleural adhesion burden, and the use of sealants or stapler buttressing—were not systematically recorded and could not be incorporated into the model. Their absence likely accounts for a substantial proportion of the unexplained variance (corrected C-statistic 0.644) and represents the primary performance ceiling for any preoperative-only prediction model. Third, FEV1 was used in absolute liters rather than as % predicted or ppoFEV1, which does not account for age, sex, and body habitus and may reduce generalizability. Fourth, the modified CCI was derived from only two components (diabetes and cerebrovascular disease) due to data availability constraints, representing only a partial approximation the of true comorbidity burden. Fifth, the comorbidity distributions observed in this cohort—including the lower hypertension and diabetes prevalence in the 60–69-year age group—may reflect surgical selection effects rather than the general population distribution. Sixth, the model was internally validated using 1000 bootstrap resamples; external validation in independent, prospectively collected RATS cohorts is required before clinical implementation. The nomogram should therefore be regarded as a preliminary preoperative risk stratification tool pending such validation. Seventh, the comparison with the Pompili ESTS and Brunelli variable sets involved re-fitting in our cohort rather than application of the original published coefficients, and does not constitute true external benchmarking of those scoring systems.

## 5. Conclusions

In this retrospective cohort study of 1185 patients undergoing robot-assisted thoracic surgery for primary lung cancer, male sex and impaired preoperative pulmonary function (FEV1) were identified as the strongest independent predictors of prolonged air leak (PAL, ≥5 days), with higher BMI exerting a modest protective effect. Age was not a significant linear predictor in the multivariable model; however, restricted cubic spline analysis revealed a significant nonlinear association, with risk increasing more steeply beyond approximately 70 years of age. This nonlinear pattern was reproducible across 3-knot and 4-knot specifications and under a more stringent PAL definition (≥7 days), supporting its robustness as an exploratory finding.

A nomogram integrating these preoperative variables demonstrated moderate discrimination (corrected C-statistic 0.644) and favorable calibration, with decision curve analysis confirming net clinical benefit over treat-all and treat-none strategies across a range of clinically relevant threshold probabilities. Risk stratification identified a threefold difference in observed PAL rates between the low- and high-risk strata. These findings highlight the importance of preoperative pulmonary function assessment and sex-specific risk awareness in patients undergoing RATS for lung cancer. External validation in independent cohorts is warranted before this tool is considered for routine clinical implementation.

## Figures and Tables

**Table 1:** Baseline Characteristics of Patients Undergoing Robotic Lung Cancer Resection, Stratified by Age Group.

Variable	<60 y (*n* = 549)	60–69 y (*n* = 457)	≥70 y (*n* = 179)	*p* Value
**Demographics**				
Male sex, No. (%)	187 (34.1)	181 (39.6)	84 (46.9)	0.006
Ever smoker, No. (%)	72 (13.1)	99 (21.7)	41 (22.9)	<0.001
**Pulmonary function**				
FEV1, mean (SD), L	2.7 (0.6)	2.3 (0.5)	2.1 (0.6)	<0.001
FEV1/FVC, mean (SD), %	81.1 (7.0)	77.8 (8.5)	75.9 (9.1)	<0.001
DLCO (SB), mean (SD), mmol/min/kPa	6.7 (1.7)	5.8 (1.5)	5.4 (1.4)	<0.001
**Anthropometric**				
BMI, mean (SD), kg/m^2^	24.1 (3.6)	23.9 (3.3)	24.1 (2.9)	0.7
**Comorbidities**				
Modified CCI, median [IQR]	0 [0–0]	0 [0–0]	0 [0–1]	<0.001
Hypertension, No. (%)	143 (26.0)	53 (11.6)	78 (43.6)	<0.001
Coronary artery disease, No. (%)	42 (7.7)	24 (5.3)	16 (8.9)	0.168
Diabetes mellitus, No. (%)	56 (10.2)	24 (5.3)	38 (21.2)	<0.001
**Surgical characteristics**				
Lobectomy, No. (%)	243 (44.3)	262 (57.3)	120 (67.0)	<0.001
Tumor size (CT), mean (SD), mm	15.7 (9.6)	18.7 (9.9)	21.8 (10.3)	<0.001
**Outcome**				
PAL (≥5 days), No. (%)	36 (6.6)	36 (7.9)	26 (14.5)	0.003

Values are mean (SD), median [IQR], or No. (%). Between-group comparisons used Kruskal–Wallis tests for continuous variables and chi2/Fisher exact tests for categorical variables. Abbreviations: BMI, body mass index; CCI, Charlson Comorbidity Index; DLCO, diffusing capacity of the lung for carbon monoxide; FEV1, forced expiratory volume in 1 s; FVC, forced vital capacity; IQR, interquartile range; PAL, prolonged air leak; SD, standard deviation.

**Table 2:** Multivariable Firth’s Penalized Logistic Regression for Prolonged Air Leak (PAL ≥ 5 days; *n* = 1185; 98 events).

Variable	OR (95% CI)	*p* Value
Age (per year)	1.00 (0.97–1.02)	0.86
**Male sex (vs. female)**	**3.29 (1.96–5.54)**	**<0.001**
Modified CCI = 1 (vs. 0)	0.88 (0.44–1.63)	0.7
Modified CCI = 2 (vs. 0)	1.66 (0.17–8.03)	0.61
**BMI (per 1 kg/m^2^)**	**0.91 (0.85–0.98)**	**0.01**
**FEV1 (per 1 L)**	**0.50 (0.33–0.77)**	**<0.001**
Lobectomy (vs. segmentectomy)	1.30 (0.84–2.05)	0.24
Ever smoker (vs. never)	1.08 (0.61–1.86)	0.79

Firth’s penalized logistic regression was used to reduce bias from sparse outcomes (events per variable = 14.0). Bold values indicate *p* < 0.05. All continuous variables are modeled as linear terms; see Table 3 for nonlinearity assessment. Abbreviations: BMI, body mass index; CCI, Charlson Comorbidity Index; CI, confidence interval; FEV1, forced expiratory volume in 1 s; OR, odds ratio; PAL, prolonged air leak.

**Table 3:** Restricted Cubic Spline Nonlinearity Tests for Age, FEV1, and BMI.

Variable	Knots	LR chi2	*p* for Nonlinearity	ΔAIC	Adjustment
Primary analysis (PAL ≥ 5 days, minimally adjusted)					
**Age**	**3**	**7.28**	**0.007**	**2.18**	**Age + BMI**
FEV1	3	1.18	0.278	−0.95	FEV1 + Age
**BMI**	**3**	**4.93**	**0.026**	**−0.65**	**BMI + Age**
Sensitivity analysis 1 (fully adjusted model)					
**Age**	**3**	**7.29**	**0.007**	**1.57**	**All covariates**
FEV1	3	1.16	0.281	−1.98	All covariates
**BMI**	**3**	**4.93**	**0.026**	**−0.96**	**All covariates**
Sensitivity analysis 2 (PAL ≥ 7 days, minimally adjusted)					
**Age**	**3**	**6.44**	**0.011**	**0**	**Age + BMI**
FEV1	3	N/A	>0.999	−1.49	FEV1 + Age
BMI	3	4.08	0.043	−0.68	BMI + Age

Nonlinearity was assessed using likelihood ratio tests comparing the RCS model (3 knots at 5th/50th/95th percentiles; Age: 42/60/73 years, FEV1: 1.52/2.38/3.49 L, BMI: 19.0/23.8/29.5 kg/m^2^) to the corresponding linear model. ΔAIC = AIClinear − AICspline; positive values indicate improved fit with the spline model; |ΔAIC| < 2 indicates marginal improvement. Bold *p* values indicate significant nonlinearity (*p* < 0.05). N/A: LRT statistic was negative (spline did not improve fit over linear model); *p* reported as >0.999. Abbreviations: AIC, Akaike information criterion; BMI, body mass index; FEV1, forced expiratory volume in 1 s; LR, likelihood ratio; PAL, prolonged air leak; RCS, restricted cubic spline.

## Data Availability

The data presented in this study are available upon request from the corresponding author. The data are not publicly available due to privacy restrictions.

## Supplementary Materials

The following supporting information can be downloaded at: https://www.mdpi.com/article/10.3390/cancers18101612/s1, Table S1: Four-Knot Restricted Cubic Spline Sensitivity Analysis for Nonlinearity of Age, FEV_1_, and BMI with PAL Risk (PAL ≥ 5 Days); Table S2: Multivariable Firth’s Penalized Logistic Regression for Prolonged Air Leak Using the ≥7-Day Definition (n = 1185; Events = 39).
