# Supplementary material for: Risk Factors and Nonlinear Risk Patterns of Prolonged Air Leak After Robot-Assisted Lung Resection for Lung Cancer: A Retrospective Cohort Study
Source: Cancers (Basel). 2026 May 15;18(10):1612. doi: 10.3390/cancers18101612 (PMC13204022; doi:10.3390/cancers18101612)
Supplement: Supplementary file 1 [file cancers-18-01612-s001.zip › cancers-4284502-supplementary.pdf]

Supplementary Tables

Supplementary Table S1. Four-Knot Restricted Cubic Spline Sensitivity Analysis for Nonlinearity of Age, FEV1, and BMI with PAL Risk (PAL ≥5 Days).

Section	Variable	Knots	LR χ^2	P for nonlinearity	ΔAIC	Adjustment
**4-knot sensitivity (PAL ≥5 days, minimally adjusted)**	**Age**	**4**	**9.44**	**0.009**	**0.16**	**Age + BMI**
	**FEV1**	**4**	**N/A**	**>0.999**	**−1.02**	**FEV1 + Age**
	**BMI**	**4**	**4.66**	**0.097**	**−2.58**	**BMI + Age**
**4-knot sensitivity (PAL ≥5 days, fully adjusted)**	**Age**	**4**	**9.46**	**0.009**	**−0.12**	**All covariates**

Nonlinearity was assessed using likelihood ratio tests (LRT) comparing the 4-knot RCS model to the corresponding linear model. Four knots were placed at the 5th, 35th, 65th, and 95th percentiles: Age 42/56/64/73 years; FEV1 1.52/2.18/2.62/3.49 L; BMI 19.0/22.8/25.0/29.5 kg/m². ΔAIC = AIClinear − AICspline; positive values indicate improved fit with the spline model; |ΔAIC| <2 indicates marginal improvement. N/A: LRT statistic was negative, indicating the spline did not improve fit over the linear model; P reported as >0.999. Bold P values indicate significant nonlinearity (P <0.05). Minimally adjusted models: Age adjusted for BMI; FEV1 adjusted for Age; BMI adjusted for Age. Fully adjusted model includes all covariates (age, sex, BMI, FEV1, smoking history, modified CCI, surgery type). These results confirm that the nonlinear association of age with PAL risk is robust to the choice of knot number (3-knot primary analysis: p = 0.007; 4-knot sensitivity: p = 0.009).

Abbreviations: AIC, Akaike information criterion; BMI, body mass index; FEV1, forced expiratory volume in 1 second; LR, likelihood ratio; PAL, prolonged air leak; RCS, restricted cubic spline.

Supplementary Table S2. Multivariable Firth Penalized Logistic Regression for Prolonged Air Leak Using the ≥7-Day Definition (*n* = 1,185; Events = 39).

Variable	OR (95% CI)	P value
Age (per year)	1.03 (0.99–1.08)	0.14
Male sex (vs female)	2.13 (0.96–4.68)	0.06
Modified CCI = 1 (vs 0)	1.48 (0.60–3.26)	0.37
Modified CCI = 2 (vs 0)	4.05 (0.36–23.26)	0.22
BMI (per 1 kg/m²)	0.95 (0.85–1.05)	0.30
**FEV1 (per 1 L)**	**0.49 (0.25–0.93)**	**0.03**
Lobectomy (vs segmentectomy)	0.87 (0.44–1.72)	0.68
Ever smoker (vs never)	1.99 (0.93–4.16)	0.08

Outcome: PAL defined as chest tube duration ≥7 days. N = 1,185; Events = 39 (3.3%); EPV = 5.6. Apparent C-statistic = 0.727. Method: Firth penalized logistic regression to reduce bias from sparse outcomes. Bold values indicate P <0.05. The limited EPV (5.6) warrants cautious interpretation; these results are intended to assess directional consistency with the primary model (PAL ≥5 days) rather than to derive an independent prediction tool.

Abbreviations: BMI, body mass index; CCI, Charlson Comorbidity Index; CI, confidence interval; EPV, events per variable; FEV1, forced expiratory volume in 1 second; OR, odds ratio; PAL, prolonged air leak.
